# Commentary: ‘Levelling down’ or ‘building back fairer’? A commentary/reflection on Wright et al. (2021)

**DOI:** 10.1111/jcv2.12011

**Published:** 2021-05-19

**Authors:** Tamsin Newlove‐Delgado

**Affiliations:** ^1^ University of Exeter Medical School Exeter UK

Against a background of conflicting narratives on the benefits and harms of COVID‐19 restrictions, there is considerable investment in defining and capturing the evidence for the impact on children and young people. At times, narratives have been polarised and politicised. In the media, this is represented by the images of hand‐drawn rainbows and family time, or of locked playground gates; Martikainen and Sakki's (2021) paper on newspaper depictions notes that children were predominantly represented as ‘controlled pupils’ or ‘joyful players’, enjoying freedoms away from school.

The success of the race to evidence the impacts has to some extent been a matter of chance timing of pre‐pandemic data collection, and the ability of a research team to be fleet of foot in assembling or following up a cohort. Wright et al. (2021), published in this issue, capitalises on this existence of a previously characterised cohort (the UK Wirral Child Health and Development Study), and of a prior wave of data collection carried out just before the pandemic. The sample is small, including just over 200 children aged 11–12, and their mothers. It is also unusual in being drawn from a more socio‐economically deprived cohort than many (two‐fifths of were living in the most deprived quintile of neighbourhoods according to the Index of Multiple Deprivation), although the sample are predominantly white, meaning that again evidence is lacking on children and young people from Black and Minority Ethnic (BAME) background. The participants contributed data as part of cohort follow‐up in December 2019 to March 2020, and were then followed‐up again in June to July 2020, approximately 3 months after the lockdown restrictions began in the United Kingdom. The commentary presents a discussion of the findings in the context of the emerging evidence on trends in child and adolescent mental health over the course of the pandemic. It focuses primarily on the UK setting, and considers the implications for mental health inequalities and for services aimed at mental health promotion and treatment.

A feature of most editorials and commentaries about COVID‐19 is an acknowledgement and rehearsal of the conflicting findings of the studies which have been done. Certainly, the sparse evidence which emerged early in the pandemic reported mixed effects, finding improvements in some measures in some groups, for example, amongst secondary school pupils scoring low on school, peer and family connectedness pre‐pandemic (Widnall et al., 2020). The findings of our follow‐up to the 2017 Mental Health of Children and Young People (MHCYP) survey, in July and August 2020, provided widely reported headline figures of a rise in prevalence of probable mental disorder in 5–16 year olds from one in nine in 2017 to one in six in 2020 (Vizard et al., 2020). Other studies have covered change within the pandemic, for example, the Co‐SPACE study which recently reported deteriorations in mental health symptoms in early lockdown amongst 4–10 year olds, with less marked changes in adolescents (Waite et al., 2020).

Wright et al.'s (2021) findings support the growing evidence of a direct impact of the period of lockdown restrictions, demonstrating an increase in 11–12 year olds of self‐reported and parent‐reported depressive symptoms, and disruptive behaviour problems. They found a widening of the gap in self‐reported depression between boys and girls, and highlight what they term ‘new risk’; a greater increase in parent‐reported depression amongst those without prior symptoms. Concerning their moderator analysis, Wright et al. (2021) also note: ‘Prior to the pandemic, rates of maternal and child depression were greater in families experiencing higher deprivation, but changed only in less deprived families, raising their rates to those of the high deprivation group’. On the surface, this contradicts what we might have expected to see, which would be that the mental health impact of COVID‐19 would be more pronounced and disproportionate amongst those with existing socio‐economic vulnerabilities. Time trend cross‐cohort comparisons have tended to suggest a widening of mental health inequalities over time (Sellers et al., 2019) rather than a convergence. Waite et al. (2020) report that household income did not moderate the change in symptoms they report from Co‐Space although this was a more affluent sample. However, although Wright et al. (2021) are limited in using neighbourhood‐level indicators rather than individual socio‐economic indicators, their findings echo research into adult mental health. Early in lockdown, UK Household Longitudinal Study (UKHLS) found that those in employment experienced a greater increase in mental distress relative to previous trends, compared to those unemployed or otherwise inactive (Pierce et al., 2020). What explains this? Wright et al. (2021), in effect, compare the most deprived in their sample to all others, and point to the existing higher levels of depression in mothers and children living in these most deprived neighbourhoods. It is likely that, in a ‘levelling down’, they have been joined by those families in the quintile above who were ‘just managing' prior to the onset of COVID‐19, who were vulnerable to being tipped over the edge by reductions in hours, small profit margins in their businesses, insecure employment and a precarious hold on what they have. This COVID‐19 effect is also suggested by cross‐sectional analyses from MHCYP 2020, which found that children and young people with probable disorder were also more likely to live in households that had newly fallen into debt during the pandemic (Vizard et al., 2020).

However, this study, and many of the others described here, publish data collected 9–12 months ago. This is a blink of the eye in research and publishing timescales, but in the context of the pandemic feels more like looking through a telescope at light from stars that might no longer exist. The impacts of a full year of uncertainty, varying levels of restrictions, furlough and job losses, cycles of closures and re‐openings, home learning and returns to schools, are not yet known. There also remains a wide range of scenarios for the years ahead in public health terms, from a full recovery, to limited restrictions, and to third and fourth waves, and new pandemics. Research is catching up; Wave 2 of the MHCYP survey is in the field at the time of writing, using many identical measures to Wave 1, and Wave 3 is planned for the summer. Along with other studies, this will allow examination of within‐pandemic change, as well as providing information on whether changes are sustained, and whether the distribution of impacts has also evolved.

Nevertheless, the findings presented highlight pronounced existing inequalities, and add to the challenge of addressing them. Many developed countries do not start from a position of strength. COVID‐19 has landed against a backdrop of concerns over deteriorating child and adolescent mental health, and over access to treatment. Three successive British/English national child mental health surveys have reported no increase in the proportion of children and adolescents with clinically impairing problems in contact with Child and Adolescent Mental Health Services, with the figure stubbornly sitting at approximately one in four. Findings from MHCYP suggest a decline in mental health‐related help‐seeking during the pandemic (Vizard et al., 2020). In ‘Build Back Fairer’, Marmot et al. (2020) emphasise the record of failure to address the pre‐existing inequalities in life chances for children and young people. In a commonly used political phrase, we have not managed to ‘level up’. If we have been unable to do this, how do we cope with the even greater challenge of reversing a ‘levelling down’, and addressing the new risk described in this and other studies? As Marmot and colleagues argue, there is an opportunity here to ‘build back fairer', but it is one which requires a longer term strategy of economic investment and a focus on non‐health solutions, alongside meeting shorter‐term mental health need. Just as the response to containing COVID‐19 has been a societal one, the same will be needed for the approach to recovery.

## CONFLICT OF INTEREST STATEMENT

No conflicts declared.

## AUTHOR CONTRIBUTION

Tamsin Newlove‐Delgado was invited to provide this commentary, drafted the work and approved it for publication.
